# The Orphan Crop *Crassocephalum crepidioides* Accumulates the Pyrrolizidine Alkaloid Jacobine in Response to Nitrogen Starvation

**DOI:** 10.3389/fpls.2021.702985

**Published:** 2021-07-28

**Authors:** Sebastian Schramm, Wilfried Rozhon, Adebimpe N. Adedeji-Badmus, Yuanyuan Liang, Shahran Nayem, Traud Winkelmann, Brigitte Poppenberger

**Affiliations:** ^1^Biotechnology of Horticultural Crops, TUM School of Life Sciences, Technical University of Munich, Freising, Germany; ^2^Woody Plant and Propagation Physiology Section, Institute of Horticultural Production Systems, Gottfried Wilhelm Leibniz University Hannover, Hanover, Germany

**Keywords:** alkaloids, domestication, ebolo, genome, neglected crop, nutrients, *Yoruba bologi*, fireweed

## Abstract

*Crassocephalum crepidioides* is an African orphan crop that is used as a leafy vegetable and medicinal plant. Although it is of high regional importance in Sub-Saharan Africa, the plant is still mainly collected from the wild and therefore efforts are made to promote its domestication. However, in addition to beneficial properties, there was first evidence that *C. crepidioides* can accumulate the highly toxic pyrrolizidine alkaloid (PA) jacobine and here it was investigated, how jacobine production is controlled. Using ecotypes from Africa and Asia that were characterized in terms of their PA profiles, it is shown that the tetraploid *C. crepidioides* forms jacobine, an ability that its diploid close relative *Crassocephalum rubens* appears to lack. Evidence is provided that nitrogen (N) deficiency strongly increases jacobine in the leaves of *C. crepidioides*, that this capacity depends more strongly on the shoot than the root system, and that homospermidine synthase (HSS) activity is not rate-limiting for this reaction. A characterization of *HSS* gene representation and transcription showed that *C. crepidioides* and *C. rubens* possess two functional versions, one of which is conserved, that the *HSS* transcript is mainly present in roots and that its abundance is not controlled by N deficiency. In summary, this work improves our understanding of how environmental cues impact PA biosynthesis in plants and provides a basis for the development of PA-free *C. crepidioides* cultivars, which will aid its domestication and safe use.

## Introduction

The availability of food is dependent on a few crops only. Estimates suggest that today 15 species provide 90% of the world’s food and plant-based energy (Gruber, 2017). While major crops are widely cultivated, their productivity depends on large resource inputs, since they were bred for performance in high input cropping systems (Fess et al., 2011). In addition to the major crops, hundreds of plants exist that have served as food sources for centuries or even millennia but are minor in terms of global trade and the research attention they receive (Heywood et al., 2013; Gruber, 2017). These underutilized or orphan crops can promote better nutrition, particularly in developing regions of the world, and help to optimize land resources by cultivating soils that are marginal or unsuitable for major crop production (Pingali, 2012; Jain and Gupta, 2013).

Many orphan crops are wild or semi-wild species with crop-like traits for which neither seed supply systems exist nor cropping schemes have been established (FAO, 1999; Jain and Gupta, 2013). Moreover, tools and methodology for plant breeding and molecular genetics that could facilitate approaches for faster domestication are underdeveloped. To improve the situation, the African Orphan Crops Consortium was founded, an initiative that aims to generate genetic resources, including whole genome sequences for 101 traditional food crops with high importance for Africa (Hendre et al., 2019; Jamnadass et al., 2020). The plant list mainly contains fruit and vegetable crops, one of which is *Crassocephalum rubens*.

*Crassocephalum rubens* and its close relative *C. crepidioides* belong to the plant family Asteraceae (Pelser et al., 2007). They are leafy vegetables and medicinal plants native to tropical Africa but grow throughout tropical and sub-tropical regions of the world. Depending on the region, various names exist, which usually refer to both species. In Africa common names are Yoruban bologi, ebolo (Nigeria), or gbolo (Benin). English names are redflower ragleaf and fireweed. The plants have interesting nutritional and medicinal properties, since they are rich in minerals, essential oils, and antioxidants (Adjatin et al., 2013a; Oyebode et al., 2019).

Despite their value in Western and Central Africa, *Crassocephalum* species are not regularly cultivated but are still mainly harvested from the wild. When cultivated and repeatedly harvested yields of *C. crepidioides* can reach 25–27 t/ha of leaves and shoots per year (Grubben, 2004). These biomass gains are achieved even in marginal conditions, to which the plant is well-adapted, and thus efforts are made to promote its wider cultivation and consumption (Joshi, 2011; Adjatin et al., 2013b).

However, in addition to beneficial properties, there is evidence that *C. crepidioides* can accumulate the pyrrolizidine alkaloid (PA) jacobine (Rozhon et al., 2018). PAs are heterocyclic compounds synthesized by plants, that are part of the chemical defense system against herbivores (Macel, 2011; Wei et al., 2015). Estimates suggest that approximately 6,000 plant species worldwide, comprising 3% of all the flowering plants, produce these secondary metabolites. In particular members of the Asteraceae, Boraginaceae, Heliotropiaceae, Apocynaceae, and some genera of the Orchidaceae are PA-producers (Ober and Hartmann, 2000).

While PAs themselves are chemically unreactive, they can be bioactivated in the intestinal tract and during transit through the liver of mammals. This gives rise to highly reactive pyrroles, which are hepatotoxic and carcinogenic (Ruan et al., 2014). Venous occlusions in the liver and lung, megalocytosis, inhibition of cell division, and liver cirrhosis are signs of PA toxicity. Genotoxic effects have been reported as well, in particular for 1,2-unsaturated PAs such as jacobine (Fu et al., 2002), which is why the European Food Safety Authority applies the “Margin of Exposure” approach and recommends a dose limit of 0.007 mg PA/kg b.w./day (EFSA Panel on Contaminants in the Food Chain, 2011). In industrialized countries, in addition to intake of PA-containing herbs (when present in teas, herbal infusions, or food supplements) in particular carry-over of PAs from feed to milk of animals and from pollen to honey is of concern (Dusemund et al., 2018; Mulder et al., 2018).

Albeit their importance as food and feed contaminants and for plant defense reactions, relatively little is known about PA biosynthesis. PAs are produced from homospermidine, which is formed by homospermidine synthase (HSS), a putrescine:putrescine 4-aminobutyl-transferase that catalyzes the first dedicated and essential reaction in PA biosynthesis (Ober and Hartmann, 1999). PAs biosynthesis then proceeds by largely unknown means but certainly involving esterification of necine acids with a necine base. On the basis of the necine bases, PAs are classified into three types: the retronecine-type, the otonecine-type, and the platynecine-type and can occur in free or as *N*-oxides bound forms (Schramm et al., 2019b). The toxic potencies of these PAs vary significantly with retronecine-type PAs being most toxic for mammals (Ruan et al., 2014) and jacobine belongs to this class. It has high acute toxicity and strong mutagenic capacity and is therefore of particular concern when present in the food chain (Mulder et al., 2018).

Here, we used *C. rubens* and *C. crepidioides* ecotypes from Africa and Asia to show that jacobine is produced by different *C. crepidioides* accessions, an ability that the studied *C. rubens* accessions lacked. Evidence is provided that jacobine formation in *C. crepidioides* is dependent on shoots and is induced by nitrogen (N) deficiency, a regulatory effect that occurs down-stream of HSS. HSS encoding genes were cloned and characterized to facilitate approaches for the development of PA-free *C. crepidioides* cultivars, which are discussed.

## Materials and Methods

### Plant Material

Seeds of two *C. rubens* and one *C. crepidioides* ecotypes were obtained from the Millenium Seed Bank (MSB) at Kew Royal Botanic Gardens (Kew, United Kingdom) and have the following MSB serial numbers: *C.r.*Mali (no. 440626), *C.r*.Burkina Faso (no. 320148) and *C.c*.Nepal (no. 31170). *Crassocephalum crepidioides* accession Ilé-Ifè from Nigeria was described previously (Rozhon et al., 2018). *Crassocephalum crepidioides* accession *C.c.*Osogbo was collected in Osogbo, Nigeria and accession *C.r.*Thailand was obtained from Thailand.

### Phylogenetic Analysis

To confirm the identity of the accessions and conduct a phylogenetic analysis, the chromosomal internal transcribed spacer (Blattner, 1999) and the trnL-trnF spacer of the chloroplast (Taberlet et al., 1991) were amplified by PCR using the primer pairs trnL fwd/trnF rev and ITS-A/ITS-B, respectively (for sequences of all the primers used in this study, see Supplemental Table 2). The amplicons were purified and sequenced, and the sequences were aligned. Neighbor joining trees were calculated with MEGA X (Kumar et al., 2018).

### Analyses of Genome Size and 5-Methylcytosine Content

Absolute nuclear DNA contents (pg/2C) were determined using nuclei isolated from greenhouse-grown plants. Approximately 0.5 cm^2^ of this young leaf material was chopped in 400 μl of the nuclei isolation buffer (Kit: CyStain PI Absolute P; Sysmex Deutschland GmbH, Norderstedt, Germany) together with 0.5 cm^2^ young leaf tissue of the internal standards, i.e., *Pisum sativum* cultivar ‘Viktoria, Kifejto Borso’ (9.07 pg/2C, Genebank IPK Gatersleben acc. no. PIS 630). The resulting suspension was incubated for 2 min, filtered (30 μm Celltric filters; Sysmex), and mixed with 1,600 μl of the staining solution of the kit (CyStain PI Absolute P; Sysmex) containing propidium iodide. After 1 h dark incubation on ice, the samples were analyzed in the flow cytometer (CyFlow Ploidy Analyzer; Sysmex Partec GmbH, Münster, Germany). Measurements were taken on at least three different dates aiming at a minimum number of 2,000 particles in the main peaks of both species. Per accession, DNA contents of 3–5 seedlings were determined, each with 3–7 replicates prepared with independently collected leaf material. For the estimation of the nuclear DNA contents, the sample mean G0/G1 peak position was divided by the standard mean G0/G1 peak position and multiplied by the DNA content of the standard (pg/2C). Genome size was calculated from the DNA content according to Dolezel et al. (2003) using the conversion factor 0.978. The 5-mdC content was measured as described previously (Rozhon et al., 2008).

### Growth Conditions in Soil

For growth in soil, plants were cultivated in growth chambers (Bright Boy; CLF Plant Climatics, Wertingen) or the greenhouse at 22 ± 2°C and cycles of 16 h white light (80 μmol m^−2^ s^−1^)/8 h dark. Four commercially available substrates with different nutrient composition were used: CL ED73 and SP ED63 P (Patzer GmbH and Co. KG, Sinntal-Altengronau, Germany) as well as D400 and C700 with Cocopor^®^ (Stender AG, Schermbeck, Germany). For substrate experiments, six 4-week-old seedlings per treatment group, pre-grown on SP ED63 P substrate, were transplanted individually into 9.5-cm plastic pots containing equal volumes of the different substrates and were grown for another 8 weeks until analysis.

For the time course experiment of nitrogen starvation, 4-week-old *C.c.*Ile-Ife seedlings, pre-grown on SP ED63 P substrate, were transplanted into pots containing 160 g of Low Nutrient Pond Soil (*COMPO SANA*^®^; COMPO GmbH, Münster, Germany) and grown for another 3 weeks. To each pot an ammonium nitrate solution was then added, to obtain 150 or 500 mg N/kg substrate, respectively. Additionally, 20 ml of a basic nutrient solution (22 g/L KH₂PO_4_, 16.2 g/L MgSO_4_·7 H_2_O, 11.7 g/L CaCl_2_·2 H_2_O (only for the second application), 3.98 g/L FeSO_4_·7 H₂O, 985 mg/L MnSO_4_·H_2_O, 157 mg/L CuSO_4_·5 H_2_O, 176 mg/L ZnSO_4_·7 H_2_O, and 46 mg/L H_3_BO_3_) were supplied. The nitrate treatment started at 7 weeks post-germination and was performed two times, in 2-week intervals. Leaf material was harvested after the last nitrate treatment from 3 to 4 individual plants per treatment group (low N and high N) in 3–4 biological replicates, 7, 14, 21, and 28 days after the last fertilization and HPLC measurements were carried out.

### Hydroponic System for N and K Starvation Experiments

The hydroponic system is shown in Supplementary Figure 1. It consisted of a growth box with a size of 28 cm × 19 cm × 14 cm and a corresponding lid with six holes, equipped with mesh net pots filled with cylindrical foam sponge blocks (Du Yang, Shenzhen, China). Each hydroponic box was used for cultivation of six plants in 4-L liquid medium, which was exchanged twice a week. Constant aeration of the medium was provided with air stones attached to an air pump (Supplementary Figure 1).

For nutrient starvation experiments 3-week-old seedlings, pre-grown in SP ED63 P, were transplanted to the hydroponic tanks and cultivated in ½ MS medium (Murashige and Skoog, 1962) for another 3 weeks before ½ MS was exchanged with a modified Hoagland medium (Hoagland and Arnon, 1938). After exchange of the medium, the plants were grown for another 3 weeks in the tanks, before the PA composition was analyzed from 3 individual plants in three biological repeats.

The Hoagland medium was composed of 1.25 mM K_2_SO_4_, 2.5 mM Ca(NO_3_)_2_, 6.8 μM C_10_H_12_N_2_NaFeO_8_, 0.5 mM MgSO_4_, 0.125 mM KH_2_PO_4_, 11.56 μM H_3_BO_3_, 2.29 μM MnCl_2_, 0.19 μM ZnSO_4_, 0.05 μM CuSO_4_, and 0.09 μM Na_2_MoO_4_ dissolved in deionized water, contained 4.28 g MES (4-morpholineethanesulfonic acid), and had the pH adjusted to 5.8 with KOH. Drop-out of nitrate was achieved by replacing Ca(NO_3_)_2_ with CaCl_2_, drop-out of potassium by omitting K_2_SO_4_.

### Grafting

The grafting procedure was adapted from Melnyk (2017). Fourteen-day-old seedlings pre-grown on ½ MS medium were transferred to Petri dishes containing two layers of wet filter papers and one layer of nylon membrane. One cotyledon was removed and transverse cuts were made through the hypocotyls below the shoot apical meristem. Dissected shoots were placed on the top of respective rootstocks to assemble the grafts. After grafting, Petri dishes were sealed with parafilm and placed vertically in an incubator in 16 h light/8 h dark cycles at 22°C. After 7 days successfully grafted plants were transferred to SP ED63 P substrate. Adventitious roots of scions were removed regularly in their early stage to maintain the desired rootstock identity. Grafted plants were cultivated in substrate for another 11 weeks before harvesting leaf tissues for analyses.

### Analysis of Metabolites and Minerals in Leaf Tissue and Soil

Jacobine was measured as described previously (Rozhon et al., 2018). Quantification of total retronecine was adapted from Kempf et al. (2008) and is described in detail in the Supplementary Information. Polyamines were quantified by HPLC after derivatization with dansyl chloride (details are also given in the Supplementary Information). Nitrate was quantified by ion-pair chromatography as described previously (Schramm et al., 2019a). For analysis of minerals homogenized frozen plant material was weighed into a quartz crucible and incinerated at 500°C for 5 h. The ash was dissolved in 1 ml 1 M nitric acid and water was added to a final volume of 10 ml. For analysis of potassium, the solution was diluted 1:20 with distilled water and analyzed by cation exchange chromatography as described previously (Schramm et al., 2019a). The phosphate content was analyzed by a modified molybdate method as described in the Supplementary Information. The growth substrates were analyzed by Agrolab (Sarstedt, Germany).

### Cloning of *HSS* Genes, Recombinant HSS Expression, and Enzyme Activity Assays

For cloning *HSS* from *Senecio jacobaea*, RNA was isolated from *S. jacobaea* roots and cDNA was generated from 2 μg RNA using the RevertAid H minus first strand cDNA synthesis kit (Thermo Fisher Scientific, Waltham, MA, United States). This cDNA was used as a template for the amplification of *HSS* coding sequences using the primer pair P70/P71 (Reimann et al., 2004; for all primer sequences, see Supplementary Table 1). Cloning in pRSET-A and sequencing revealed three SjHSS versions that differed only by a few amino acids in non-conserved regions.

For cloning of *Cc*HSS, DNA was isolated from young *C. crepidioides* Ile-Ife leaves and PCR reactions performed using the degenerated primer pairs SxHSS fwd1/SxHSS rev1 and SxHSS fwd2/SxHSS rev2 that were designed to bind to the most conserved parts of known *HSS* genes of the Senecioneae. Both yielded amplicons with a size of approximately 1 kb. Sequencing confirmed homology to the HSS gene of *S. jacobaea*. Using these sequences, primers were designed for 5'-RACE (primers CcHSS 5'-RACE 1, 2, and 3) and 3'-RACE (primers CcHSS 3'-RACE 1, 2, and 3). The template cDNA was prepared as described above using RNA isolated form *C. crepidioides* Ile-Ife roots. 5'-RACE and 3'-RACE were performed as described previously (Scotto-Lavino et al., 2006a,b). The obtained sequences were deposited in the EMBL or GenBank nucleotide sequence database with the following accession numbers: *C.c.*Ile-Ife_*HSS1*, LR999447; *C.c.*Ile-Ife_*HSS2*, LR999448; *C.c*.Nepal_*HSS1*, LR999449; *C.c*.Nepal_*HSS2*, LR999450; *C.r.*Burkina_Faso_*HSS2*, MZ275249; *C.r.*Mali_*HSS2*, MZ275250; *S.j*_*HSS2*, LR999444; and *S.j*_*HSS3*, LR999445. These sequences were aligned with the sequences of HSS orthologues of *S. jacobaea* (HSS1, AJ704850), *Senecio vulgaris* (HSS, AJ251500), and *Senecio vernalis* (HSS1, AJ238623; HSS2, AJ704849) retrieved from the NIH GenBank,^1^ and a neighbor joining tree was calculated with bootstrap test (1,000 replicates) using MEGA X (Kumar et al., 2018).

The coding *HSS* sequences were cloned into the pRSET-A vector (Thermo Fisher Scientific) to produce His-tagged versions. These constructs were transformed in *Escherichia coli* BL21 and expression of His-tagged proteins induced by addition of IPTG to a final concentration of 1 mM. The proteins were purified using HisPur™ cobalt resin (Thermo Fisher Scientific) as recommended by the manufacturer. Enzymatic activity assays were carried out in 50 μl reactions containing 1 μg of recombinant HSS fusion protein, 25 μl TEA-buffer (100 mM, adjusted to pH 9.5 with phosphoric acid), 1 μl EDTA (5 mM), 1 μl DTT (50 mM), 2 μl putrescine (1 mM), 2 μl spermidine (1 mM), and 2.5 μl NAD^+^ (25 mM). Reactions were incubated for 1 h at 30°C and stopped by the addition 100 μl 2 M sodium carbonate. Polyamines were derivatized and concentrations were measured as described in the Supplementary Information.

### Quantitative PCR Analyses

For quantitative PCR (qPCRs) RNA was isolated from respective samples with the E.Z.N.A. Plant RNA Kit (Omega Bio-tek, Norcross, GA, United States), and cDNA was synthesized with the RevertAid First Strand cDNA Synthesis Kit (Thermo Fisher Scientific, Waltham, MA, United States). qPCRs were carried out with the Mastercycler RealPlex2 (Eppendorf, Hamburg, Germany) as described previously (Unterholzner et al., 2015). In brief, PCR reactions contained 2 μl cDNA, the primer pair CcHSS1+2 fwd/CcHSS rev for *HSS1*+2 analysis and a SYBR green master mix (Nippon Genetics Europe, Düren, Germany). As a reference gene *C. crepidioides* glyceraldehyde-3-phosphate-dehydrogenase C2 (*CcGAPC2*) was used (He et al., 2016) and amplified with the primer pair CcG.

### Statistical Analyses

Statistical significance was determined with one-way ANOVA followed by Tukey’s HSD tests. Significant differences at *p* ≤ 0.05 are indicated with different letters. Details on the numbers of replicates are given in the respective figure legends.

## Results

### Isolation of *Crassocephalum crepidioides* and *Crassocephalum rubens* Ecotypes That Differ in Vegetative Development and Genome Composition

Previously, we have shown that a *C. crepidioides* accession from Ilé-Ifè in Nigeria (*C.c.*Ile-Ife) can accumulate jacobine but does not form any other senecionine-type PAs (Rozhon et al., 2018). To investigate, if the ability to form jacobine is ecotype-specific and address, whether differences in jacobine accumulation between *C. crepidioides* and *C. rubens* occur, we obtained three additional *C. crepidioides* ecotypes, from Nepal (*C.c.*Nepal), Thailand (*C.c.*Thailand) and Osogbo in Nigeria (*C.c.*Osogbo), and two accessions of *C. rubens*, from Mali (*C.r.*Mali) and Burkina Faso (*C.r.*Burkina Faso). A phylogenetic analysis, based on ITS and trnL-trnF sequences, showed that, as expected, Asian and African *C. crepidioides* and *C. rubens* accessions were most strongly related (Supplementary Figures 2A,B).

A rough phenotypic comparison of these ecotypes showed that plants of *C. crepidioides* were generally larger than *C. rubens* and formed more leaves (Supplementary Figure 2C), before entering the generative stage. Moreover, the vegetative growth phase, defined as the time from seed germination to first flower buds developing, was significantly prolonged in the African *C. crepidioides* ecotypes, which took approximately 72 days until flowering and approximately 110 days until seed dispersal (Supplementary Figure 2D). In addition, the African accessions formed more leaves, which were also more serrated (Supplementary Figures 2C,D). Since the two African and Asian *C. crepidioides* ecotypes had comparable phenotypic characteristics, we focused on one African (*C.c.*Ile-Ife) and one Asian line (*C.c.*Nepal) for the subsequent work.

To compare the genetic make-up of the ecotypes the chromosome numbers and genome sizes were determined. In general, the genus *Crassocephalum* has a basic chromosome number of *n* = 10 (Oyelakin and Ayodele, 2013). *C.c.*Ile-Ife contained 40 chromosomes (Rozhon et al., 2018), which indicated tetraploidy, and was in line with previous reports (Vanijajiva and Kadereit, 2009; Oyelakin and Ayodele, 2013). The ploidy level of *C. rubens* was described as diploid (Oyelakin and Ayodele, 2013). To verify this for the additional ecotypes, chromosomes were counted in root tip cells. This showed that *C.c.*Nepal, like *C.c.*Ile-Ife, contained 40 chromosomes, whereas the two *C. rubens* ecotypes *C.r.*Mali and *C.r.*Burkina Faso contained 20 (Supplementary Figure 2E), confirming *x* = 10 and tetraploid and diploid states, respectively. In both species, chromosomes were acrocentric or submetacentric and small (Supplementary Figure 2E).

To determine the genome sizes, flow cytometry measurements were conducted. The mean 2C value of *C.c.*Ile-Ife and *C.c.*Nepal was 12.4 pg, which implies a genome size of approximately 12.1 Gbp. The genomes of the two *C. rubens* accessions *C.r.*Mali and *C.r.*Burkina Faso had 2C values of 6.1 pg (Supplementary Table 2). For a further comparison of the genome composition, the level of 5-mdC DNA methylation was determined, which impacts trait expression and plasticity. The results showed that the 2-week-old seedlings of *C. crepidioides* and *C. rubens* had a similar 5-mdC content of approximately 30 and 31.5 mol%, respectively (Supplementary Table 2).

### Nitrogen Starvation Increases Jacobine in Leaves of *Crassocephalum crepidioides*, But Not of *Crassocephalum rubens*

**Figure 1:**
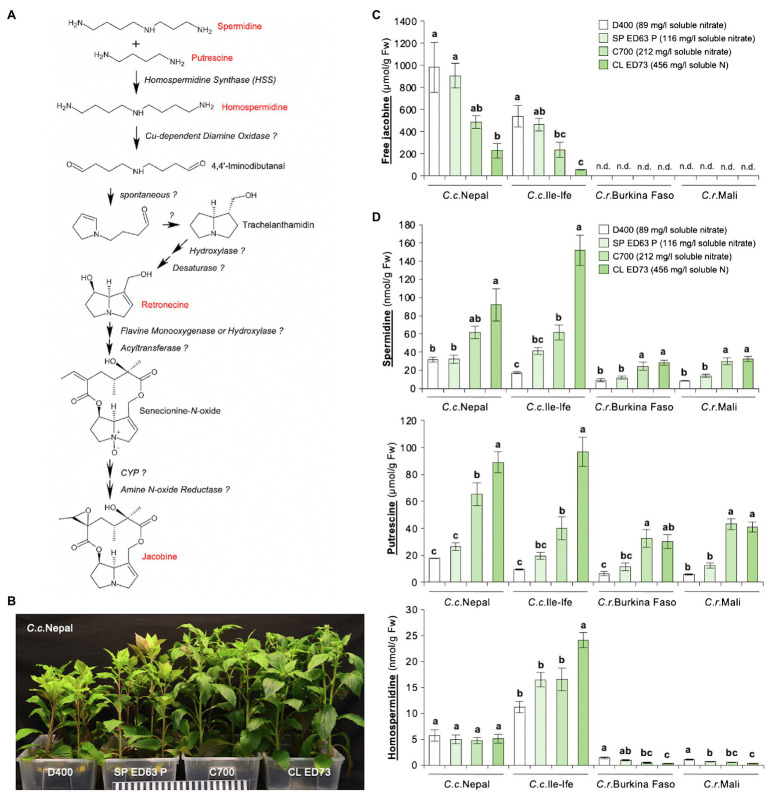
Leaves of *Crassocephalum crepidioides*, but not of *Crassocephalum rubens*, accumulate jacobine, which is promoted by growth on low nitrate substrate. **(A)** Tentative PA biosynthetic pathway required for the biosynthesis of jacobine (modified from Schramm et al., 2019b). PAs and up-stream precursors analyzed are marked in red. Unknown enzymes are labeled with? **(B)** Representative 8-week-old plants of the *Crassocephalum crepidioides* ecotype Nepal, grown on the indicated growth substrates in long-day growth conditions at 22 ± 2°C. **(C)** Amount of free jacobine in leaves of 8-week-old plants of *Crassocephalum crepidioides* and *Crassocephalum rubens* (grown as in **B**) measured from six plants. Nutrient analyses showed that D400 and SP ED63 P are low in nitrate, C700 has medium nitrate and CL ED73 has high nitrate levels (soluble nitrate concentrations are shown in the legend; for details on all nutrients see Supplementary Table 1). **(D)** Amounts of the polyamines spermidine, putrescine, and homospermidine in leaves of plants grown as in **(B)**. The columns and bars represent the average and standard error of six independent replicates. Statistically significant difference at *p* ≤ 0.05 of results between substrates (within individual species) is indicated with different letters and was determined with one-way ANOVA followed by Tukey’s HSD test [jacobine: *C.c.*Nepal (ANOVA *F*3,20 = 7.251, *p* = 0.002), *C.c.*Ile-Ife (ANOVA *F*3,20 = 11.199, *p* = 0.0002); spermidine: *C.c.*Nepal (ANOVA *F*3,20 = 8.482, *p* = 0.001), *C.c.*Ile-Ife (ANOVA *F*3,20 = 37.897, *p* < 0.0001), *C.r.*Burkina Faso (ANOVA *F*3,19 = 9.375, *p* = 0.0005), *C.r.*Mali (ANOVA *F*3,20 = 23.154, *p* < 0.0001); putrescine: *C.c.*Nepal (ANOVA *F*3,20 = 31.576, *p* < 0.0001), *C.c.*Ile-Ife (ANOVA *F*3,20 = 31.307, *p* < 0.0001), *C.r.*Burkina Faso (ANOVA *F*3,19 = 8.016, *p* = 0.001), *C.r.*Mali (ANOVA *F*3,20 = 42.433, *p* < 0.0001); homospermidine: *C.c.*Nepal (ANOVA *F*3,20 = 0.226, *p* = 0.877), *C.c.*Ile-Ife (ANOVA *F*3,20 = 10.980, *p* = 0.0002), *C.r.*Burkina Faso (ANOVA *F*3,19 = 16.822, *p* < 0.0001), *C.r.*Mali (ANOVA *F*3,20 = 14.753, *p* < 0.0001)].

Jacobine is synthesized in the PA biosynthetic pathway, where the formation of homospermidine from the polyamines spermidine and putrescine by HSS is the first essential reaction (Figure 1A). Since previously we had found that growth conditions impact levels of jacobine in *C.c.*Ile-Ife (Rozhon et al., 2018), we speculated that the substrate type and in particular the supply with nutrients may be relevant. To test this, four different growth substrates with different nutrient content were used. Plants grown on the substrates D400 and SP ED63 P, which are low in nitrate (Supplementary Table 3), developed clear symptoms of N deficiency (Ueda et al., 2017; Yang et al., 2018), including stunted growth, chlorotic leaves and anthocyanin accumulation (Figure 1B). In line, an analysis of leaf tissues showed that these plants were nitrate-deficient, whereas they had similar contents of potassium (K) and phosphorus (P; Supplementary Figure 3). The N deficiency was correlated with an enhanced accumulation of free jacobine in leaves of the two *C. crepidioides* lines *C.c.*Nepal and *C.c.*Ile-Ife. Interestingly, both *C. rubens* accessions did not form jacobine on any of the substrates used, with jacobine levels being below the detection limit of 4 nmol/g Fw (Figure 1C).

To assess, if the jacobine increase may result from increases in up-stream precursors, we measured the levels of spermidine, putrescine, and homospermidine in the same plants. This showed that the concentrations of none of these polyamines increased in response to low N, but, on the contrary, spermidine and putrescine were clearly decreased. Interestingly, while unable to form jacobine, *C. rubens* ecotypes did form homospermidine, albeit at low concentrations, indicating that it is not HSS expression or activity, which restricts jacobine synthesis in *C. rubens* (Figure 1D).

**Figure 2:**
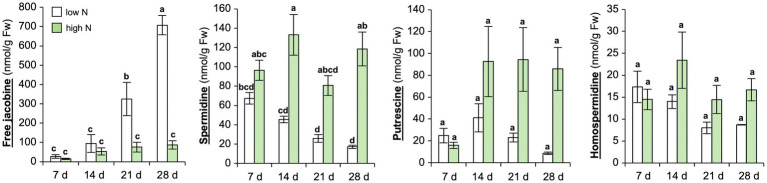
Nitrogen starvation yields strong increases of jacobine in *Crassocephalum crepidioides* leaves. *C.c.*Ile-Ife plants, grown in low nutrient pond soil, were fertilized with ammonium nitrate either at a low (150 mg N/kg DW) or at a high dose (500 mg N/kg DW). The nitrate treatment started at 7 weeks post germination and was performed two times, in 2-week intervals. Leaf samples were taken from 3 to 4 plants per treatment group at the indicated time-points after the last ammonium nitrate addition and concentrations of free jacobine, spermidine, putrescine, and homospermidine were measured by HPLC. The columns and bars represent the average and standard error of 3–4 independent biological replicates. Statistically significant difference of results between time-points and N levels at *p* ≤ 0.05 is indicated with different letters and was determined with one-way ANOVA followed by Tukey’s HSD test (jacobine: ANOVA *F*7,23 = 28.356, *p* < 0.0001; spermidine: ANOVA *F*7,20 = 12.029, *p* < 0.0001; putrescine: ANOVA *F*7,20 = 3.880, *p* = 0.008; homospermidine: ANOVA *F*7,20 = 1.984, *p* = 0.109).

To further verify, if N depletion induces jacobine formation in *C. crepidioides* leaves, a time course experiment was carried out with *C.c.*Ile-Ife. Plants were grown in a low nutrient pond soil supplemented with nutrients. N was added to this growth substrate in form of ammonium nitrate (NH_4_NO_3_) in two doses, namely a total of 150 mg N/kg DW or 500 mg/kg DW, which were added in aliquots, the first after planting of the seedlings and the second 2 weeks later. Subsequently, samples were collected at 7, 14, 21, and 28 days after the last ammonium nitrate addition and jacobine and polyamine precursors were measured in leaf tissues. This confirmed that free jacobine concentrations strongly increased, whereas levels of homospermidine and spermidine decreased, when the supply of nitrate dropped (Figure 2). Therefore, in summary these data substantiate that N starvation induces jacobine accumulation in *C. crepidioides* and suggest that homospermidine formation is not rate-limiting for this reaction.

### An Impact of N Depletion on Jacobine Accumulation Occurs Down-Stream of HSS

**Figure 3:**
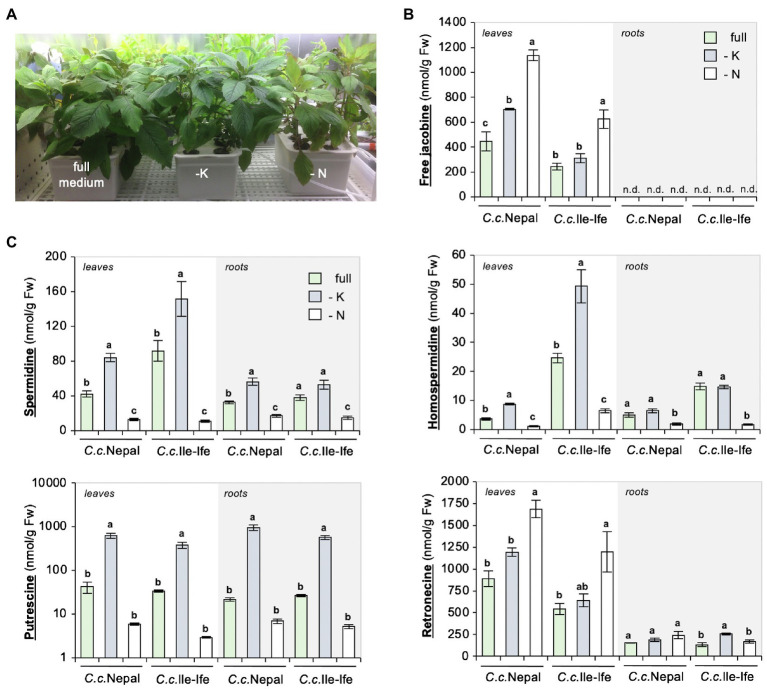
Drop-out of nitrogen increases jacobine and total retronecine, but not polyamines in leaves of hydroponically grown *Crassocephalum crepidioides* plants. Three-week-old, soil-grown *C.c.*Nepal and *C.c.*Ile-Ife plants were transferred to a hydroponic system, adapted for 3 weeks to growth in the hydroponic conditions and then transferred to either full, or potassium (-K) or nitrogen (-N) drop-out medium. After 3 weeks of growth in the drop-out medium, leaves or roots were analyzed separately from three individual plants. **(A)** Picture of representative *C.c.*Nepal plants. **(B)** Levels of free jacobine. n.d. (not detected): below the detection limit of 4 nmol/g Fw. **(C)** Levels of spermidine, putrescine, homospermidine and total retronecine. The columns and bars represent the average and standard error of 3 independent biological replicates. Statistically significant difference of results within organs of the individual species at *p* ≤ 0.05 is indicated with different letters and was determined with one-way ANOVA followed by Tukey’s HSD test [free jacobine: *C.c.*Nepal leaves (ANOVA *F*2,6 = 46.821, *p* = 0.0002), *C.c.*Ile-Ife leaves (ANOVA *F*2,6 = 16.451, *p* = 0.004); spermidine: *C.c.*Nepal leaves (ANOVA *F*2,6 = 99.300, *p* < 0.0001), *C.c.*Ile-Ife leaves (ANOVA *F*2,6 = 27.509, *p* = 0.001), *C.c.*Nepal roots (ANOVA *F*2,6 = 51.917, *p* = 0.0002), *C.c.*Ile-Ife roots (ANOVA *F*2,6 = 25.216, *p* = 0.001); homospermidine: *C.c.*Nepal leaves (ANOVA *F*2,6 = 181.942, *p* < 0.0001), *C.c.*Ile-Ife leaves (ANOVA *F*2,6 = 39.788, *p* = 0.0003), *C.c.*Nepal roots (ANOVA *F*2,6 = 13.364, *p* = 0.006), *C.c.*Ile-Ife roots (ANOVA *F*2,6 = 91.586, *p* < 0.0001); putrescine: *C.c.*Nepal leaves (ANOVA *F*2,6 = 57.970, *p* = 0.0001), *C.c.*Ile-Ife leaves (ANOVA *F*2,6 = 41.215, *p* = 0.0003), *C.c.*Nepal roots (ANOVA *F*2,6 = 44.225, *p* = 0.0003), *C.c.*Ile-Ife roots (ANOVA *F*2,6 = 76.987, *p* < 0.0001); retronecine: *C.c.*Nepal leaves (ANOVA *F*2,6 = 24.150, *p* = 0.001), *C.c.*Ile-Ife leaves (ANOVA *F*2,6 = 5.923, *p* = 0.038), *C.c.*Nepal roots (ANOVA *F*2,6 = 2.696, *p* = 0.146), *C.c.*Ile-Ife roots (ANOVA *F*2,6 = 12.140, *p* =0.008)].

There is little evidence that N deficiency can induce PA biosynthesis as yet. In *Senecio* species, increased NPK fertilization decreased PA concentrations in shoots (Vrieling et al., 1993; Vrieling and van Wijk, 1994; Hol et al., 2003; Kirk et al., 2010); however, which nutrient accounted for these effects had remained unknown. To conclusively assess, whether it is N depletion that causes jacobine accumulation, a hydroponic system was adopted for the species. The system (shown in Supplementary Figure 1 and Figure 3A) allows for plant growth in soil-free conditions, targeted delivery of compounds and individual sampling of shoot and root material.

*C.c.*Nepal and *C.c.*Ile-Ife plants were pre-grown for 3 weeks in growth substrate and then transferred either to tanks containing liquid medium with all required nutrients (full) or tanks containing drop-out medium, which lacked either potassium (-K) or nitrate (-N). A separate analysis of young leaf and root tissues showed that free jacobine significantly increased in response to N deficiency in leaves of both accessions but was below the detection limit of 4 nmol/g Fw in roots, uncovering organ specific differences. K deficiency had a significant impact on jacobine amounts in leaves of *C.c.*Nepal, but not of *C.c.*Ile-Ife (Figure 3B).

In line with the results from soil-grown plants, the levels of polyamines decreased in response to N deficiency. In contrast, K deficiency increased polyamines, in particular, putrescine (Figure 3C; please note the logarithmic scale), which has also been described for other plant species (Richards and Coleman, 1952; Liu et al., 2015). As opposed to free jacobine, polyamines are present in similar quantities in leaves and roots, showing that it is not a shortage of these up-stream precursors that impairs jacobine formation in roots of *C. crepidioides*.

To further define the sites of N deficiency impact, the levels of retronecine, a product formed later in PA biosynthesis (Figure 1A), was determined in the same samples. The method used allows a quantification of both free and (in retronecine-derived PAs) bound retronecine. The results of the analysis showed that, in the leaves of both ecotypes, total retronecine levels were increased by about 2-fold when grown in medium without nitrate, which was correlated with an increase of jacobine by about 2.5-fold (Figures 3B,C). In contrast, jacobine was not detectable in roots, while total retronecine was also present, albeit at lower concentrations. Importantly, total retronecine was responsive to N depletion in shoots, but not in roots, which implies that the regulatory impact is shoot-specific. In summary, the impact of nitrate deficiency on jacobine synthesis occurs down-stream of homospermidine, likely at the level or down-stream of retronecine formation.

### Jacobine Formation in *Crassocephalum crepidioides* Requires Shoot Organs

In principle, PAs can be present in all plant organs, but in species of the Asteraceae family, such as *S. vulgaris* (common groundsel), they are thought to be synthesized exclusively in roots, and then, as *N*-oxides, transported *via* the phloem to the shoots (Hartmann et al., 1989). Since jacobine was undetectable in roots (Figure 3B), we intended to test, if the root system is necessary for jacobine formation in *C. crepidioides*. To enable this, we established a grafting system for *Crassocephalum* and grafted shoots of the jacobine-producer *C.c.*Ile-Ife onto rootstocks of *C.r.*Mali, which does not form jacobine. Scions of 2-week-old *C.c.*Ile-Ife plants were splice grafted onto rootstocks of *C.r.*Mali and *C.c.*Ile-Ife, as a control. Grafting of *C.r.*Mali onto rootstocks of *C.c.*Ile-ife and *C.r.*Mali generated grafts with an opposite composition.

**Figure 4:**
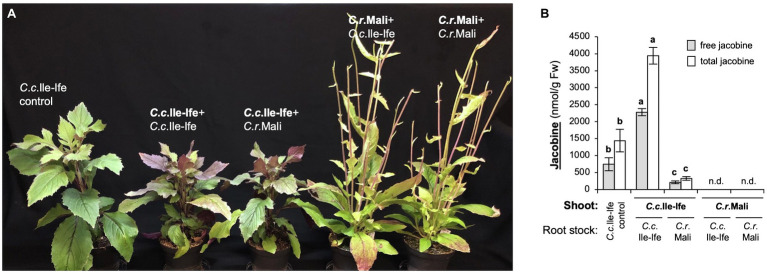
Jacobine accumulation in *Crassocephalum crepidioides* relies on its shoot organs. Two-week-old scions of *C.c.*Ile-Ife were grafted onto root stocks of *C.r.*Mali and vice versa and maintained in tissue culture for 1 week for establishment. Grafted plants, grafting controls (scions united with rootstocks of the same species) and non-grafted *C.c.*Ile-Ife plants (as a control) where then transferred to SP ED63 (low nitrate) substrate and grown for another 11 weeks in standard growth conditions, before free and total jacobine was measured in shoots. **(A)** Pictures of representative plants. The grafting combinations are named with the shoot in first position (in bold letters) and the root stock in second position. **(B)** Levels of free and total jacobine in nmol/g Fw. Plants are labeled like in A (shoot donor in bold). The columns and bars represent the average and standard error of 6–17 independent replicates. Statistically significant difference at *p* ≤ 0.05 of results is indicated with different letters and was determined with one-way ANOVA followed by Tukey’s HSD test (free jacobine: ANOVA *F*2,30 = 164.719, *p* < 0.0001; total jacobine: ANOVA *F*2,30 = 130.194, *p* < 0.0001). n.d. (not detected): below the detection limit of 4 nmol/g Fw.

Grafted plants showed signs of stress, such as increased anthocyanin accumulation and growth retardation, as compared to non-grafted *C. crepidioides* plants (Figure 4A). This was a grafting reaction, since control grafts (shoots joint with root stocks of the same species) showed the same phenotypes as the inter-species combinations. Thus, functional grafts had been generated and measurements of jacobine (both free and total, which includes its *N*-oxide) from leaf tissues were carried out. These showed that in leaves of the *C.c.*Ile-Ife + *C.c.*Ile-Ife control grafts, as compared to non-grafted *C. crepidioides* plants levels of both free and total jacobine were significantly increased, showing that grafting, in addition to causing (other) stress-response reactions, also stimulated jacobine production. The *C. rubens* control grafts, which combined *C.r.*Mali shoots with *C.r.*Mali root stock, also showed stress symptoms, but did not produce jacobine.

Importantly, when the *C.c.*Ile-Ife roots-stock was replaced with a *C.r.*Mali root stock, jacobine levels were clearly decreased as compared to the *C.c.*Ile-Ife + *C.c.*Ile-Ife control grafts, providing evidence that it is the shoot system that is central for jacobine formation in *C. crepidioides*, but also showing that the rootstock contributes. This was confirmed by analyzing the grafting combinations in the opposite direction: when *C. rubens* contributed the shoot, jacobine was not formed, even when a *C. crepidioides* rootstock was avaiflable in the *C.r.*Mali + *C.c.*Ile-Ife graft (Figure 4B). Therefore, it is mainly shoot organs that are required for jacobine formation in *C. crepidioides*.

### In *Crassocephalum crepidioides* Two *HSS* Genes Exist, Which Are Mainly Expressed in Roots

To further investigate, if a regulatory impact of N deficiency on jacobine synthesis occurs down-stream of HSS, we characterized *HSS* genes of *C. crepidioides* and *C. rubens*. For this purpose, degenerated primers targeting conserved *HSS* sequences were used and *HSS* from all four accessions were cloned. This revealed that *C. rubens* and *C. crepidioides* both contain two *HSS* variants, one of which, *HSS1*, was identical in both species and a second, *HSS2*, which slightly differed (Supplementary Figure 4). A phylogenetic analysis of the cloned HSSs with HSS orthologues cloned from other members of the Senecioneae, showed that, as expected, *Crassocephalum* HSSs were more related with each other than with other HSSs (Supplementary Figure 5).

**Figure 5:**
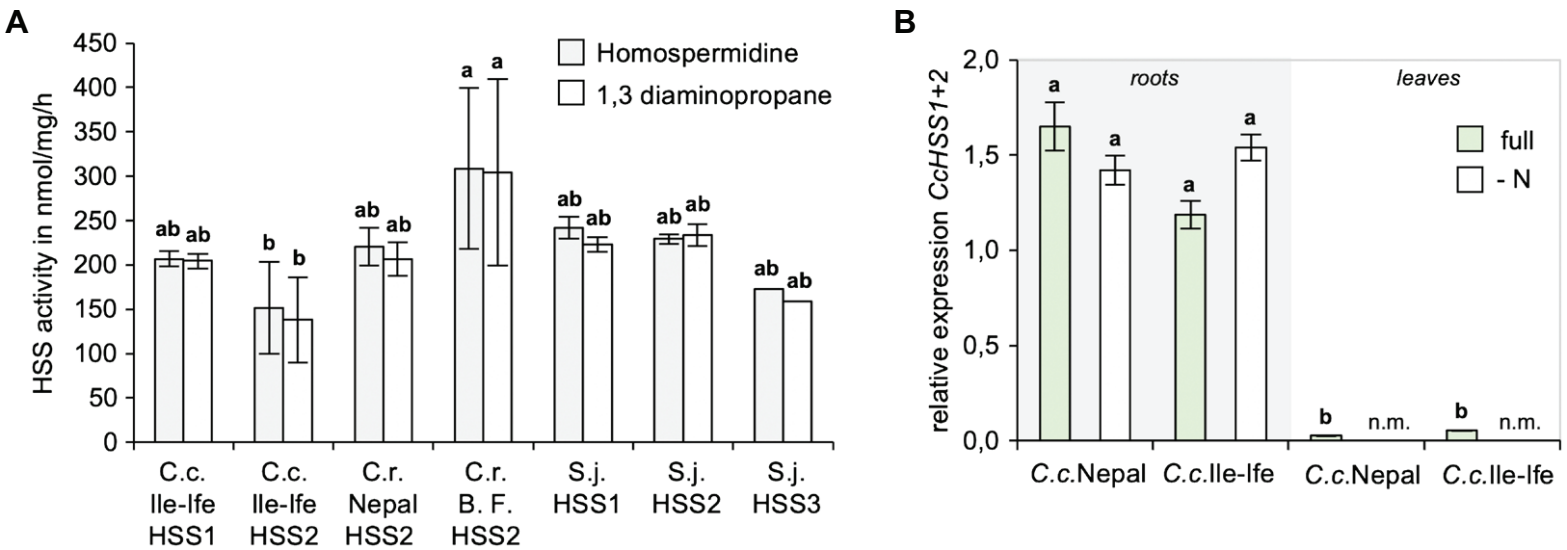
HSS1 and HSS2 of *Crassocephalum crepidioides* are enzymatically active and mainly expressed in roots. **(A)** Comparison of *in vitro* activities of HSS1 and HSS2 of *Crassocephalum crepidioides* with HSS2 of *Crassocephalum rubens* and HSS versions of *Senecio jacobaea*. Recombinant, His-tagged proteins were used in *in vitro* enzyme assays with spermidine and putrescine as substrates and analyzing formation of spermidine and the by-product 1,3-diaminopropane by HPLC. The columns and bars represent the average and standard error of 1–5 independent biological replicates. Letters above the bars indicate *p* < 0.05, ANOVA followed by Tukey’s HSD test (homospermidine: ANOVA *F*6,13 = 4.406, *p* = 0.012; by-product: ANOVA *F*6,13 = 4.648, *p* = 0.010). **(B)** qPCR analyses of *CcHSS1*+2 expression in leaves of *Crassocephalum crepidioides* plants grown hydroponically either in full or in nitrate drop-out medium. The average and standard deviation of 3 independent biological replicates. Letters above the bars indicate *p* < 0.05, ANOVA followed by Tukey’s HSD test (ANOVA *F*5,11 = 15.781, *p* = 0.0001). n.m., not measured.

To study, if the identified HSSs are enzymatically active and compare their activities to the HSS orthologues of the PA-producer *S. jacobaea* (ragwort), His-tagged versions of all HSSs were cloned, expressed in *E. coli*, recombinant proteins were purified and *in vitro* enzyme assays with spermidine and putrescine as substrates were carried out. This showed that all the enzymes produced homospermidine, as well as a homospermidine by-product 1,3-diaminopropane, with similar activities *in vitro* (Figure 5A).

To analyze, if expression of the HSS encoding genes of *C. crepidioides* may be controlled by N starvation, expression analyses from hydroponically grown plants of *C.c.*Nepal and *C.c.*Ile-Ife were performed. As a housekeeping gene *GAPC2* was chosen, a gene that is routinely used as reference (He et al., 2016), which was cloned from *C.c.*Ile-Ife. Since *HSS1* and *HSS2* are highly similar, the primers used for qPCRs target both *HSS* versions. The results showed that in both ecotypes of *C. crepidioides HSS1*+2, while being predominantly expressed in roots were also detectable in shoots, albeit at low levels (Figure 5B). We intended to determine *HSS1*+2 transcripts levels also following N starvation, however from N-depleted shoots hardly any RNA with sufficient quality could be extracted, making qPCR analyses impossible. From N-starved roots, qPCRs could be performed and showed that *HSS1*+2 transcript did not respond to N withdrawal (Figure 5B), providing further support to the notion that *HSS* regulation does not account for the increases in jacobine levels triggered by N starvation in *C. crepidioides*.

## Discussion

*Crassocephalum rubens* and *C. crepidioides* are African orphan crops that are used as leafy vegetables and medicinal plants in Sub-Saharan Africa. *Crassocephalum crepidioides* is particularly popular in Nigeria and, compared to its diploid close relative *C. rubens*, it brings clear benefits in terms of yields, since it has a prolonged vegetative growth phase and produces higher amounts of biomass (Adjatin et al., 2013b). These abilities could relate to the tetraploid nature of the species, since polyploidy can delay flowering and increase plant vigor (Comai, 2005; Mayfield et al., 2011; Wei et al., 2019). However, also natural variation in flowering time exists in *C. crepidioides*: the African ecotypes analyzed flowered significantly later than the Asian ones.

In addition to impacting trait plasticity, a well-described effect of polyploidy is a larger accumulation of secondary metabolites, including alkaloids (Dhawan and Lavania, 1996; Gaynor et al., 2020), and here we show that *C. crepidioides* can synthesize the PA jacobine, an ability that *C. rubens* lacks, at least in the conditions that were tested. While this may be an evolutionary advantage for the species (te Beest et al., 2012), it is an anti-nutritional trait when *C. crepidioides* is used as a leafy vegetable or medicinal plant. Therefore, as an essential step in the domestication of this wild crop, jacobine requires removal, either through specific agricultural practice, which may be difficult to implement, or genetically, through breeding.

In this study, we show that N starvation strongly increased jacobine amounts in *C. crepidioides* shoots, which are the consumed plant parts. In general, it is well-established that nutrient deficiency, like other environmental stress types, can increase the production of secondary metabolites such as anthocyanins (Yang et al., 2018), and also *C. crepidioides* hyper-accumulates these pigments in response to N deficiency. In addition, there was already evidence that nutrient availability can affect alkaloid concentrations. For example, in the legume *Lupinus angustifolius* (lupin), which produces the quinolizidine alkaloid (QA) lupanine, K shortage strongly increased lupanine levels (Gremigni et al., 2001). These increases were also seen in ‘sweet lupin’ varieties, that were bred to be QA-free, a breeding success achieved in the 1970s, which enabled for the species to become established as a crop (Kaiser et al., 2020).

While PA biosynthesis in *C. crepidioides* did not strongly respond to a deficiency in K, it clearly increased when N deficiency was induced. Such an N impact on PA levels appears to be species-specific. In *Crotalaria*, a subtropical genus of the Fabaceae, N shortage in the growth substrate did not alter PA levels in above-ground organs. However, it was shown that N depletion promoted the formation of root nodules, which host N-fixing symbiotic bacteria, and that PAs were formed specifically in these nodules (Irmer et al., 2015). In the genus *Senecio*, which like *Crassocephalum* belongs to the Asteraceae tribe Senecioneae, PA concentrations in leaves were decreased in response to NPK fertilization (Hol et al., 2003; Kirk et al., 2010); however, it remained elusive, which nutrients confer these effects. Here, we show that in *C. crepidioides* it is a deficiency in N that increases jacobine and that the regulatory impact occurs down-stream of HSS, since homospermidine concentrations were independent from N supply. In fact, levels of polyamines were decreased by N withdrawal, which could result from an increased flux through the biosynthetic pathway.

Levels of retronecine, which is formed later in PA biosynthesis, did also increase in response to N deficiency. However, since total retronecine was measured, we cannot map the sites of impact on PA biosynthetic enzymes. PA biosynthesis is largely unresolved today, but it is clear that some required enzymes are encoded by members of large gene families, making their identification difficult. One approach that could be used is RNA-sequencing of plants challenged with stimuli that induce PA biosynthetic gene expression. However, a *de novo* assembly of RNA-Seq data is very challenging and will be facilitated, once a reference genome for *C. rubens* exists. This will also benefit research and breeding activities for other members of the Asteraceae, since, although the Asteraceae is the largest family of vascular plants, which comprises 8% of all plant species (Anderberg et al., 2007), only five members have been sequenced yet: horseweed (*Erygeron canadensis*; Peng et al., 2014), sunflower (*Helianthus annuus*; Badouin et al., 2017), artichoke (*Cynara scolymus*; Scaglione et al., 2016), lettuce (*Lactuca sativa*; Reyes-Chin-Wo et al., 2017), and sweet wormwood (*Artemisia annua*; Shen et al., 2018). None of these species belong to the Senecioneae, which is the largest tribe of the Asteraceae (Pelser et al., 2007). Genome sizes in the Senecioneae strongly fluctuate from 0.79–52.3 pg/2C (Vitales et al., 2019), and here it is shown that *C. rubens* has a size of approximately 6.1 pg/2C with 2*n* = 2*x* = 20 chromosomes and *C. crepidioides* a size of approximately 12.3 pg/2C with a chromosome number of 2*n* = 4*x* = 40.

Genome information is also needed for fast-track molecular breeding approaches, such as genome editing, which could be used to remove jacobine from *C. crepidioides*, for example, through *HSS* mutation. To facilitate this approach, we cloned HSS variants from *C. rubens* and *C. crepidioides* and show that HSS is likely present in two copies, both of which are active, at least *in vitro*. Interestingly, although the two *HSS* versions of *C. crepidioides* are mainly expressed in roots, homospermidine was also detected in leaves, which suggests that it can be translocated from root to shoot organs. While homospermidine was detectable in both *C. crepidioides* and *C. rubens*, it was present in much lower abundance in leaves of the latter, which could, at least in part, account for the inability of *C. rubens* to form jacobine. In addition, also other shoot capacities appear to restrict jacobine formation in *C. rubens*, since grafted plants that contained *C. rubens* shoots were unable to form the PA, even in the presence of a homospermidine-producing *C. crepidioides* root stock. These abilities may include the production and/or mobilization of additional jacobine precursors, and there are reports from other non-PA-producing plants that a translocation of PA *N*-oxides through the phloem is a limiting factor in PA formation (Hartmann et al., 1989).

Grafting of *C. rubens* with *C. crepidioides* produced vital plants: they grew and generated leaves, shoots, and flowers, showing that even in an inter-species combination the grafting was successful. Both intra- and inter-species grafts showed signs of stress, including stunted growth and anthocyanin accumulation, and this is regularly observed (Melnyk and Meyerowitz, 2015; Gaut et al., 2019). Stress symptoms such as anthocyanin accumulation were linked with the jacobine accumulation in both grafted and N-depleted *C. crepidioides* plants, and it is therefore possible that the induction of jacobine formation is part of a general stress responsive pathway utilized to accumulate PAs.

Avoiding N shortage is one conceivable strategy to produce low-PA *C. crepidioides* plants; however, this may be difficult, if not impossible, to consistently implement in agricultural systems. Also, since an impact of other factors on PA production is conceivable, jacobine concentrations would need to be monitored to ensure safe use and this will constitute a major challenge for producers in Sub-Saharan Africa. Alternatively, jacobine-free lines could be generated, for example, through genetic removal of HSS, and here we generate first results on HSS sequence, expression, and regulation, to facilitate this approach. Very recently, it has been shown that this approach could be feasible since in *Symphytum officinale HSS* mutation yielded PA-free plants (Zakaria et al., 2021). However, it is unclear, if PA-free *C. crepidioides* varieties may bear disadvantages, such as reduced herbivore resistance, and while the fact that *C. rubens* appears to be PA-free may speak against it, this would require testing. The establishment of tools of molecular genetics, such as transformation techniques and CRISPR/Cas9 genome editing are now required, to address these biological questions and accelerate the development of PA-free *C. crepidioides* cultivars, for the benefit of domestication and safe use of this wild crop.

## Data Availability Statement

The datasets presented in this study can be found in online repositories. The names of the repository/repositories and accession number(s) can be found in the article/Supplementary Material.

## Author Contributions

SS, WR, and BP planned and designed the research. SS performed the grafting. SS and WR did the DNA methylation analysis, established the hydroponics system, carried out the metabolite analyses, and did the HSS characterization and qPCR analyses. YL took part in the *HSS* cloning. SN took part in the N depletion experiments on pond soil. AA-B performed the developmental phenotyping. TW conducted the flow cytometry measurements. BP wrote the paper with input from SS and WR. All authors contributed to finalizing the article and approved the submitted version.

## Conflict of Interest

The authors declare that the research was conducted in the absence of any commercial or financial relationships that could be construed as a potential conflict of interest.

## Publisher’s Note

All claims expressed in this article are solely those of the authors and do not necessarily represent those of their affiliated organizations, or those of the publisher, the editors and the reviewers. Any product that may be evaluated in this article, or claim that may be made by its manufacturer, is not guaranteed or endorsed by the publisher.

## Footnotes

^1^ www.ncbi.nlm.nih.gov/genbank
