# Supplementary material for: The Orphan Crop Crassocephalum crepidioides Accumulates the Pyrrolizidine Alkaloid Jacobine in Response to Nitrogen Starvation
Source: Front Plant Sci. 2021 Jul 28;12:702985. doi: 10.3389/fpls.2021.702985 (PMC8355542; doi:10.3389/fpls.2021.702985)
Supplement: Supplementary file 1 [file Data_Sheet_1.PDF]

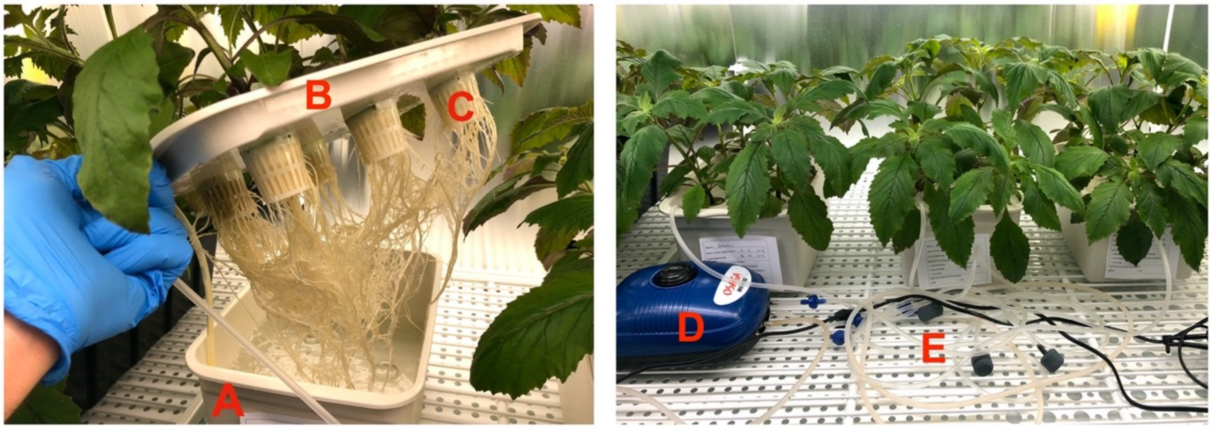

SUPPLEMENTARY FIGURE 1. Setup of the hydroponic system. Left: *C. crepidioides* plants grown in a representative growth box (A). The box was closed with a lid (B) equipped with 6 mesh net pots (C) filled with foam sponges through which the plants grew. The system allows for compounds to be added or removed to the liquid growth medium on demand and above and below ground organs to be harvested separately. Right: An air pump (D) over air stones (E) supplied the tanks with constant aeration.

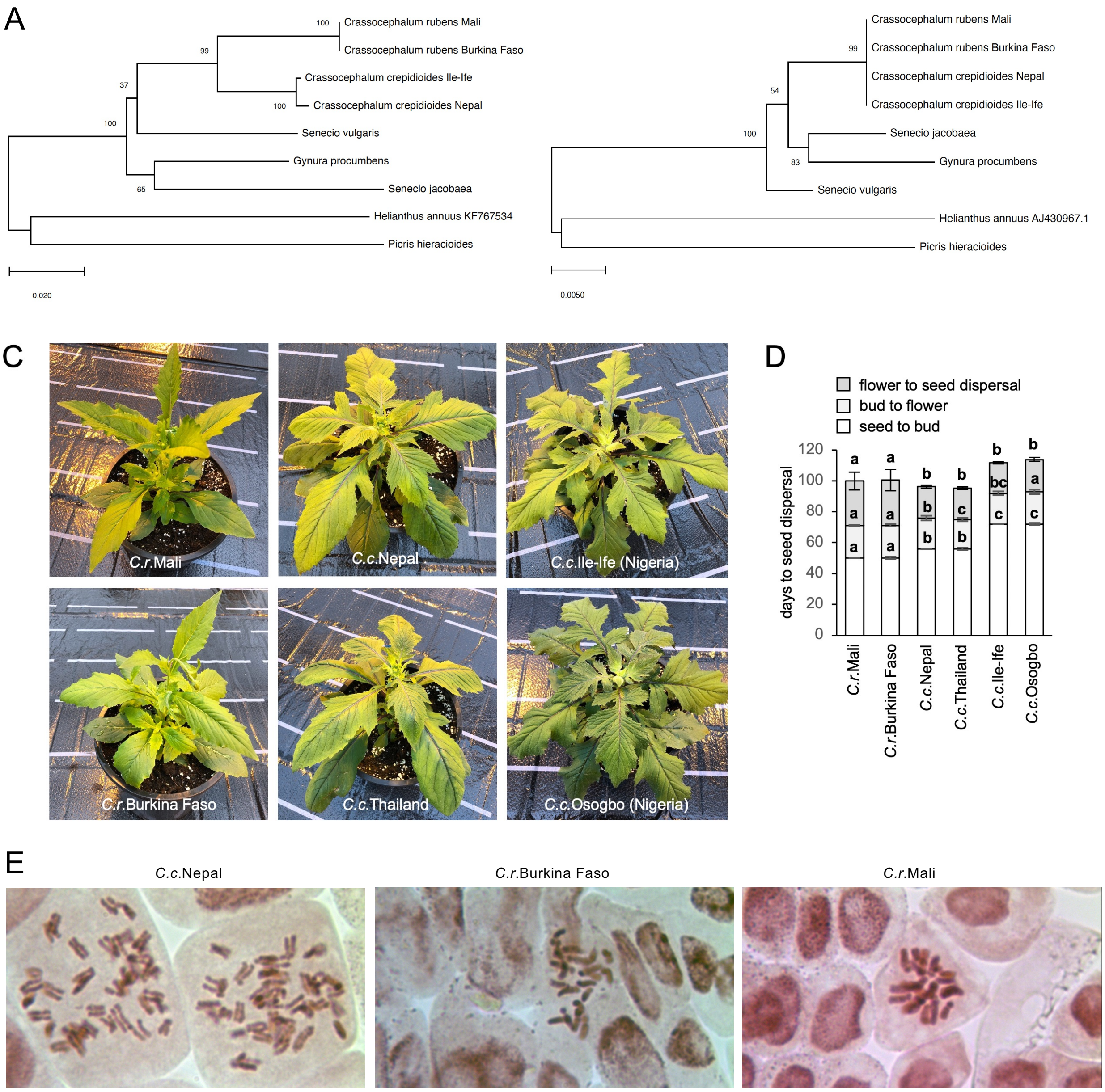

SUPPLEMENTARY FIGURE 2. Characterization of *C. crepidioides* and *C. rubens* accessions used in this study. **(A,B)** Phylogenetic tree based on ITS (A) and the *trnL-trnF* intergenic region (B). For both A and B Neighbour-Joining trees bootstrap test (500 replicates) of nucleotide sequences were generated in MEGA X. **(C)** Developmental characteristics of 8-week-old plants of *C. crepidioides* and *C. rubens* accessions from Africa and Asia grown in the greenhouse in 16 hours light/ 8 hours dark cycles at 22 +/- 2° C. Left: *C. rubens* ecotypes; center: *C. crepidioides* ecotypes from Asia; right: *C. crepidioides* ecotypes from Africa. Representative plants are shown. **(D)** Evaluation of the time from germination to flowering. Plants were grown as in A and the days to first bud development, first flower development and first seed dispersal were counted. The average and standard deviation of 10 plants is shown. Statistically significant difference at $P \leq 0.01$ between ecotypes is indicated with different letters and was determined with one-way ANOVA with a post-hoc Tukey HSD test (flower-seed dispersal: ANOVA $F_{5,132}=31.25$, $p<0.0001$; bud-flower: ANOVA $F_{5,139}=15.59$, $p<0.0001$; seed-bud: ANOVA $F_{5,54}=4338$, $p<0.0001$). **(E)** Chromosomes of *C. crepidioides* C.c.Nepal and *C. rubens* *C.c.*Burkina-Faso and C.c.Mali stained with aceto-orcein and visualized in root tip cells using light microscopy, with a magnification of 100-fold.

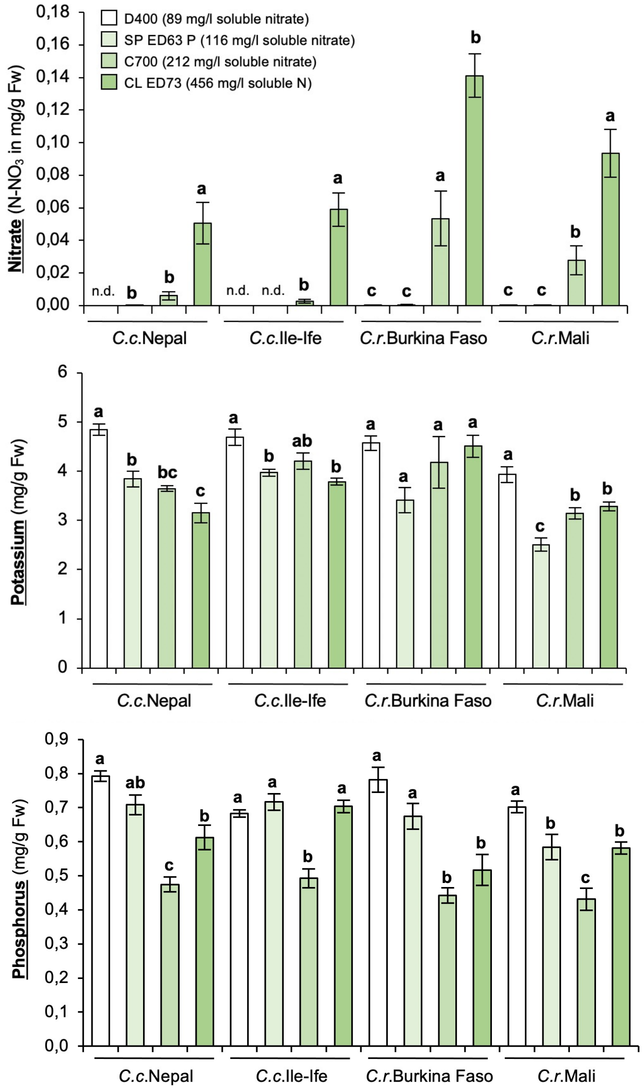

SUPPLEMENTARY FIGURE 3. Nutrient content in leaves of plants grown on the four different growth substrates used. *C. crepidioides* and *C. rubens* plants were grown for 8 weeks in cycles of 16 hours light/ 8 hours dark at 22 +/-2° C in the indicated growth substrates, leaves were harvested, nutrients were extracted and analyzed in 6 independent biological replicates. Statistically significant difference at $P \leq 0.05$ of results within accessions is indicated with different letters and was determined with one-way ANOVA with a post-hoc Tukey HSD test (nitrate: *C.c.*Nepal (ANOVA $F_{3,20} = 14.088$, $p < 0.0001$), *C.c.*Ile-Ife (ANOVA $F_{3,20} = 31.765$, $p < 0.0001$), *C.r.*Burkina Faso (ANOVA $F_{3,20} = 38.679$, $p < 0.0001$), *C.r.*Mali (ANOVA $F_{3,20} = 26.354$, $p < 0.0001$); potassium: *C.c.*Nepal (ANOVA $F_{3,20} = 24.184$, $p < 0.0001$), *C.c.*Ile-Ife (ANOVA $F_{3,20} = 9.378$, $p = 0.0004$), *C.r.*Burkina Faso (ANOVA $F_{3,20} = 2.753$, $p = 0.069$), *C.r.*Mali (ANOVA $F_{3,20} = 21.109$, $p < 0.0001$); phosphorus: *C.c.*Nepal (ANOVA $F_{3,20} = 26.410$, $p < 0.0001$), *C.c.*Ile-Ife (ANOVA $F_{3,20} = 24.413$, $p < 0.0001$), *C.r.*Burkina Faso (ANOVA $F_{3,20} = 17.600$, $p < 0.0001$), *C.r.*Mali (ANOVA $F_{3,20} = 16.022$, $p < 0.0001$)).

```
                                1                                                                              80
      C.c./C.r._HSS1     (1)   MAASNKEAIDSARSNVFKESESLEGTCSKIGGYDFNNGINYSKLLKSMVSTGFQASNLGDAMSIVNQMLDWRLSHEQSPE
    C.c.Ile-Ife_HSS2     (1)   MAASNKEAIDSARSNVFKESESLEGTCSTIGGYDFNNGINYSKLLKSMVSTGFQASNLGDAMSIVNQMLDWRLSHEQTPE
      C.c.Nepal_HSS2     (1)   MAASNKEAIDSARSNVFKESESLEGTCSTIGGYDFNNGINYSNLLKSMVSTGFQASNLGDAMSIVNQMLDWRLSHEQTPE
           C.r._HSS2     (1)   MAASNKEAIDSARSNVFKESESLEGTCSTIGGYDFNNGINYSKLLKSMVSTGFQASNLGDAMSIVNQMLDWRLSHEQTPE
           Consensus     (1)   MAASNKEAIDSARSNVFKESESLEGTCSTIGGYDFNNGINYSKLLKSMVSTGFQASNLGDAMSIVNQMLDWRLSHEQTPE
                                81                                                                            160
      C.c./C.r._HSS1    (81)   NCSEEEKKSRESVKCKVFLGFTSNLISSGVRETICYLAQHRMVDVLVTTTGGIEEDFIKCLASTYKGKFSLPGAELRSKG
    C.c.Ile-Ife_HSS2    (81)   NCSEEEKKNRESVKCKVFLGFTSNLISSGVRETICYLAQHRMVDVLVTTTGGIEEDFIKCLASTYKGKFSLPGAELRSKG
      C.c.Nepal_HSS2    (81)   NCSEEEKKNRESVKCKVFLGFTSNLISSGVRETICYLAQHRMVDVLVTTTGGIEEDFIKCLASTYKGKFSLPGAELRSKG
           C.r._HSS2    (81)   NCSEEEKKNRESVKCKVFLGFTSNLISSGVRETICYLAQHRMVDVLVTTTGGIEEDFIKCLASTYKGKFSLPGAELRSKG
           Consensus    (81)   NCSEEEKKNRESVKCKVFLGFTSNLISSGVRETICYLAQHRMVDVLVTTTGGIEEDFIKCLASTYKGKFSLPGAELRSKG
                                161                                                                           240
      C.c./C.r._HSS1   (161)   LNRIGNLIVPNDNYIKFEDWIIPIFDQMLIEQKTKNVLWTPSRIIARLGKEINNESSYLYWAYKNNIPVFCPSLTDGSIG
    C.c.Ile-Ife_HSS2   (161)   LNRIGNLIVPNDNYIKFEDWIIPIFDQMLIEQKTKNVLWTPSRIIARLGKEINNESSYLYWAYKNNIPVFCPSLTDGSLG
      C.c.Nepal_HSS2   (161)   LNRIGNLIVPNDNYIKFEDWIIPIFDQMLIEQKTKNVLWTPSRIIARLGKEINNESSYLYWAYKNNIPVFCPSLTDGSLG
           C.r._HSS2   (161)   LNRIGNLIVPNDNYIKFEDWIIPIFDQMLIEQKTKNVLWTPSGIIARLGKEINNESSYLYWAYKNNIPVFCPSLTDGSLG
           Consensus   (161)   LNRIGNLIVPNDNYIKFEDWIIPIFDQMLIEQKTKNVLWTPSRIIARLGKEINNESSYLYWAYKNNIPVFCPSLTDGSLG
                                241                                                                           320
      C.c./C.r._HSS1   (241)   DMLYFHSVSNPGPGLVVDIVQDVIAMDNEAVHASPQKTGIIILGGGLPKHHICNANMMRNGADYAVFINTAQEYDGSDSG
    C.c.Ile-Ife_HSS2   (241)   DMLYFHSVSNPGPGLVVDIVQDVIAMDNEAVHASPQKTGIIILGGGLPKHHICNANMMRNGADYAVFINTAQEYDGSDSG
      C.c.Nepal_HSS2   (241)   DMLYFHSVSNPGPGLVVDIVQDVIAMDNEAVHASPQKTGIIILGGGLPKHHICNANMMRNGADYAVFINTAQEYDGSDSG
           C.r._HSS2   (241)   DMLYFHSVSNPGPGLVVDIVQDVIAMDNEAVHASPQKTGIIILGGGLPKHHICNANMMRNGADYAVFINTAQEYDGSDSG
           Consensus   (241)   DMLYFHSVSNPGPGLVVDIVQDVIAMDNEAVHASPQKTGIIILGGGLPKHHICNANMMRNGADYAVFINTAQEYDGSDSG
                                321                                              369
      C.c./C.r._HSS1   (321)   ARPDEAVSWGKISSSGKAVKVHCDATIAFPLLVAETFAAKKEKGSKVNG
    C.c.Ile-Ife_HSS2   (321)   ARPDEAVSWGKISSSGKAVKVHCDATIAFPLLVAETFAAKKEKGSKVNG
      C.c.Nepal_HSS2   (321)   ARPDEAVSWGKISSSGKAVKVHCDATIAFPLLVAETFAAKKEKGSKVNG
           C.r._HSS2   (321)   ARPDEAVSWGKISSSGKAVKVHCDATIAFPLLVAETFAAKKEKGSKVNG
           Consensus   (321)   ARPDEAVSWGKISSSGKAVKVHCDATIAFPLLVAETFAAKKEKGSKVNG
```

SUPPLEMENTARY FIGURE 4. Amino acid alignment of HSS variants cloned from *C. crepidioides* (*C.c.*Nepal and *C.c.*Ile-Ife) and *C. rubens* (*C.r.*Mali). Identical amino acids are in black, non-identical are in white with black background.

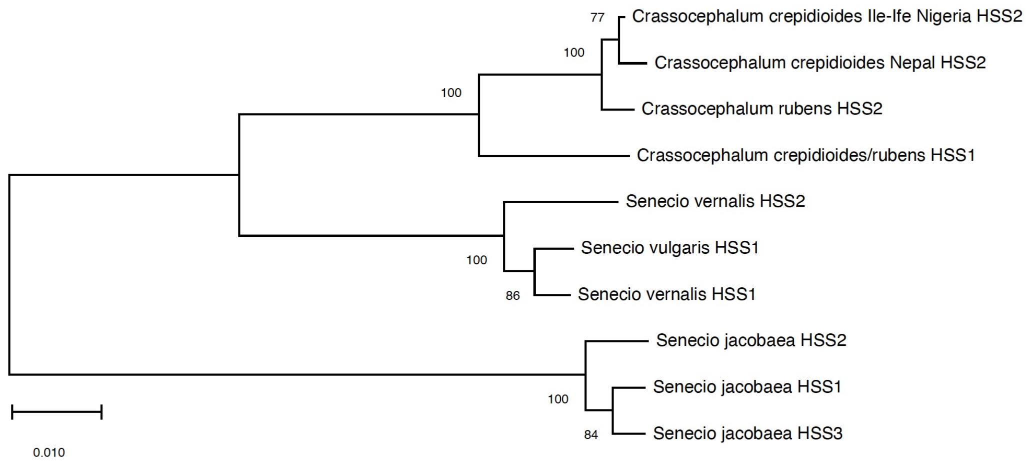

SUPPLEMENTARY FIGURE 5. Phylogenetic analysis of *Crassocephalum* HSSs. Neighbor-Joining tree with bootstrap test (500 replicates) of HSS amino acid sequences from *C. crepidioides*, *C. rubens*, *Senecio vulgaris* and *S. jacobaea*. For *S. jacobaea* a sequence retrieved from the Gene bank and two sequences obtained in this study were included. Evolutionary distances were computed using the JTT matrix and are in the units of the number of amino acid substitutions per site. Evolutionary analyses were conducted in MEGA X.

Name	Sequence 5´-3´	Used for
**trnL fwd**	CGAAATCGGTAGACGCTACG	Phylogenetic analysis (Taberlet et al., 1991)
**trnF rev**	ATTTGAACTGGTGACACGAG	
**ITS-A**	GGAAGGAGAAGTCGTAACAAGG	Phylogenetic analysis (Blattner, 1999)
**ITS-B**	CTTTTCCTCCGCTTATTGATATG	
**P70**	ATATCCATGGGAGAGACCAACAAATCAGCC	Cloning of SjHSS (PMID 15466410)
**P71**	ATATGCGGCCGCGAAACCCATTGAGTTTAGATGCTTTCT	
**SxHSS fwd1**	GAGACTATTYGTTATCTAACTCARCATCGNATG	Degenerated primers binding to a conserved region of *HSS* genes of the Senecioneae
**SxHSS rev1**	AGCAATGGGAATGCAATAGTTGCMTCACA	
**SxHSS fwd2**	GGTGGYATTGAGGARGATTTCATAAAATG	
**SxHSS rev2**	GCGAAATCTGCRCCATTACGCATCAT	
**CcHSS 5´-RACE 1**	TCGATATAGTACAAGGTGAGCACTG	5´-RACE for sequencing of the 5´-Region of the *CcHSS* gene
**CcHSS 5´-RACE 2**	TGTATGTGCTTGCTAGGCATTTTA	
**CcHSS 5´-RACE 3**	TTCCATAGCATCACCAAGATTAGA	
**CcHSS 5´-RACE 1**	TATACTTCCATTCAGTTTCCAATCC	3´-RACE for sequencing of the 5´-Region of the *CcHSS* gene
**CcHSS 5´-RACE 2**	TGGGATAATCATTTTAGGAGGAGGG	
**CcHSS 5´-RACE 3**	GCGAACATGATGTGTAATGGTGCT	
**CcHSS gDNA fwd**	GAGTAACAACAACAACTCACACTTAATAAAG	Amplification of full length *Crassocephalum* HSS genomic sequences
**CcHSS gDNA rev**	TAAAGCTGAAATACATTGAAGGAAGAG	
**CcHSS cDNA fwd**	ACTTGGATCCCATATGGCCGCGTCAAACAAAGAAGCA	Amplification of HSS cDNA
**CcHSS cDNA rev**	TATAGCGGCCGCTCAGAATTCATAGCCATTCACTTTAGATC CCTTCTC	
**CcHSS1+2 geno fwd**	AAAATACATATCATTGTCGTTTGAACA	Amplification of HSS Introns
**CcHSS1+2 geno rev**	AAGTAACATAAGTTTTAATGAAGCATATC	
**CcHSS1+2 fwd**	CGATATCGTACAAGATGTGATAG	Expression analysis of CcHSS1+HSS2 by qPCR
**CcHSS1+2 fwd**	CTAAAATGATTATCCCGGTCTTC	
**CcGAPC2 cDNA fwd**	GTGATTAACGACAGATTTGGAATTG	
**CcGAPC2 cDNA rev**	TTAATGGCAGCCTTGATCTGCTC	
**CcGAPC2 qPCR fwd**	CACTGGAGCTGCCAAGGCTGTT	
**CcGAPC2 qPCR rev**	GGAAGGCCATTCCGGTCAATTTCC	

SUPPLEMENTARY TABLE 1. Primers used in this study.

Accession	DNA content in pg/2C (n; m)	Genome size in Gbp	Chromosome no.	5-mdC content in mol% (n)
***C.c.*Nepal**	12.42 ± 0.08 (5; 32)	12.15 ± 0.08	40	30.02 ± 1.03 (5)
***C.c.*Ile-Ife**	12.35 ± 0.10 (3; 13)	12.08 ± 0.10	40	29.98 ± 1.21 (5)
***C.r.*Burkina Faso**	6.12 ± 0.03 (5; 35)	5.82 ± 0.04	20	31.45 ± 1.54 (4)
***C.r.*Mali**	6.09 ± 0.02 (3; 21)	5.80 ± 0.04	20	31.70 ± 1.34 (5)

SUPPLEMENTARY TABLE 2. Size and DNA methylation level of the *C. crepidioides* and *C. rubens* genomes. Genome size was calculated from the DNA content according to Dolezal et al., 2003 using the conversion factor 0.978. The number of biological replicates (n) and measurements (m) is shown. 5-mdC content was measured in DNA of 2-week-old seedlings grown in 16 hour light/ 8 hour dark cycles in soil and calculated as mol (5-mdC) per mol (dC+5-mdC) from n biological repeats.

Analyte	Unit	**D400**	**SPED63P**	**C700**	**CL ED73**
**Sum parameters**					
Volume weight, wet	g/l	372	323	300	453
pH		5,3	4,9	5,9	4,9
Salinity	mg/l	1161	1126	1727	3564
Conductivity	μS/cm	591	660	1090	1490
**Plant available nutrients (CAT extract)**					
N (soluble)	mg/l	89	116	212	456
N (Ammonia)	mg/l	1,9	31,2	1,5	2,3
N (Nitrate)	mg/l	86,7	84,6	210	454
P_2O_5 (Phosphate)	mg/l	30	30	28	44
K_2O (Potassium)	mg/l	211	154	295	408
Magnesium	mg/l	94	100	65	218
Sodium	mg/l	25	8	42	24
Aluminium	mg/l	4,2	5,6	2,9	7,0
Sulphur	mg/l	170	230	190	380
Iron	mg/l	30,0	42,0	58,2	77,9
Manganse	mg/l	20	10	22	17
Copper	mg/l	1,0	0,8	0,7	2,1
Boron	mg/l	0,18	0,11	0,34	0,42
Zinc	mg/l	1,6	1,6	1,3	3,1
**Further nutrients and parameters**					
Calcium	mg/l	1008	987,1	1014	1890
Molybdenum	mg/l	<0,05	<0,05	<0,05	<0,05
Chloride	mg/l	26	<8	75	172
Carbonate	%	0,4	<0,2	0,5	0,2

SUPPLEMENTARY TABLE 3. Nutrient content of the growth substrates used, determined by a commercial analysis.

SUPPLEMENTARY MATERIALS AND METHODS

Quantification of Retronecine

Quantification of total retronecine was adapted from (Kempf et al., 2008). In brief, 20 mg of finely ground frozen plant material was extracted with 1 ml of 20 mM citric acid containing 5 µg of heliotrine as an internal standard (which is converted to heliotridine during sample workup) at 1400 rpm, 25°C for 30 min. After centrifugation at 13000 rpm for 10 min, the supernatant was transferred to a new tube and 200 µl 2-propanol was added. Subsequently, 10 mg of zinc dust was added followed by shaking at 1400 rpm, 25°C for 30 min. The supernatant was loaded onto a Chromabond SA (100 mg) strong cation exchange SPE column preconditioned with 1 ml methanol followed by 1 ml 50 mM sulfuric acid. The columns were washed with 1 ml water and 1 ml methanol prior elution with 1 ml ammoniated methanol (3 ml 25% ammonia solution in 50 ml methanol). The eluate was immediately dried in a SpeedVac. After drying 100 µl lithium aluminum hydride solution (1 M in THF) was added and the samples were incubated at room temperature for 30 min. Subsequently, 500 µl *tert*-butyl methyl ether and 50 µl of 4 M sodium hydroxide solution were added and the samples were mixed at 1400 rpm, 25°C for 10 min. The organic phase was collected and the residue was extracted one more with *tert*-butyl methyl ether. The combined organic phases were dried in a SpeedVac. The residue was mixed with 50 µl *N*-methyl-*N*-(trimethylsilyl)-trifluoroacetamide and incubated at 40°C for 30 min. The resulting solutions were transferred into micro-inserts and analyzed by GC-MS.

The GC-MS system consisted of a CP-8400 autosampler and a 431-GC gas chromatograph connected to a 210-MS mass spectrometer (Varian, Palo Alto, CA, USA). A VF-5ms 30 m × 0.25 mm capillary column with 0.25 µm film thickness (Agilent, Santa Clara, CA, USA) was installed in the GC-MS system. Helium was used as carrier gas at a flow rate of 1 ml/min. The injector was set to 200°C and injections (1 µl sample) were performed in the splitless mode. The temperature program of the column oven started with an isothermal step at 60°C for 1 min. Then the temperature was raised linearly within 4.5 min to 150°C at a rate of 20°C/min. Next, the temperature was increased within 6 min to 210°C at a rate of 10°C/min. Finally, the temperature was raised within 3 min to 300 °C at a rate of 30°C/min prior returning it to the initial conditions. The transfer line was operated at 180°C, the ion trap at 150°C and the manifold at 40°C. MS spectra were recorded from 10 to 11.5 min from m/z 50 to 400. Retronecine appeared at a retention time of 10.9 min and the fragment with m/z 157 was used for quantification while the fragments with m/z 317, 303, 227 and 73 served as qualifier ions. The internal standard heliotridine appeared at a retention time of 10.5 min and the fragment with m/z 183 was used for quantification while the fragments with m/z 299, 93 and 73 served as qualifier ions.

Quantification of Polyamines

Homogenized frozen plant material (20-30 mg, weighed to the nearest 0.1 mg) was transferred into a reaction tube and 400 µl ice-cold 5% perchloric acid and 100 µl internal standard (40 µM 1,7-diaminoheptane and 600 µM L-2-aminoadipic acid [for quantification of amino acids; see below] in 5% perchloric acid) were added and shaken at 1400 rpm and 4°C for 1 h. After centrifugation (12000 g, 5 min) the supernatant was collected. For analysis of polyamines, 100 µl supernatant was mixed with 100 µl 2 M sodium carbonate and 200 µl derivatisation reagent (100 mg dansyl chloride dissolved in 10 ml acetone) and incubated at 60°C for 1 h. Excess reagent was quenched by addition of 200 µl 1 M sodium glutamate pH 9.5 and incubation at room temperature for 15 min. The reaction was extracted twice with 500 µl toluene. The combined organic extracts were washed once with 500 µl 2 M sodium carbonate solution prior evaporation in a vacuum concentrator to dryness.

The residue was dissolved in 200 µl 60% ACN and analyzed by HPLC using a Shimadzu 10A system (Shimadzu, Kyoto, Japan).

The HPLC system consisted of a SCL-10A system controller, a FCV-10AL valve for eluent selection, a LC-10AT pump equipped with DGU-14A inline degasser, a SIL-10A autosampler, a CTO-10ASvp column, a Nucleodur 100-5 C18ec 125 x 4,6 mm HPLC column (Machery-Nagel, Düren, Germany), a SPD-10A UV detector and a RF-10Axl fluorescence detector. Eluent A was 10% (v/v) ACN in water and eluent B pure ACN. Elution was performed at a flow rate of 1 ml/min with a gradient starting with 50% A and 50% B. The concentration of B was linearly increased within 23 min to 75% and subsequently within 10 min to 90%. Finally, the concentration of B was reduced to the initial conditions within 1 min and the column equilibrated for 7 min prior injection of the next sample. The injection volume was 25 µl, the column oven temperature set to 25°C and the fluorescence detector was operated at an excitation wavelength of 340 nm and an emission wavelength of 510 nm.

Quantification of Phosphorous and Ammonia

An appropriate volume of sample (CAT extract or plant material ash dissolved in nitric acid) was transferred into a 1 cm cuvette and water added to a total volume of 800 µl. Subsequently, 200 µl freshly prepared molybdate reagent (2 ml 4 M hydrochloric acid, 1.5 ml 2.5% ammonium molybdate and 1.5 ml 10% ascorbic acid) was added. The mixture was incubated at room temperature for 2 h prior measurement of the absorption at 820 nm. Standards containing 0-3 mg/l P (in form of potassium dihydrogen phosphate) were used for establishing the calibration curve.

CAT extract (500 µl) was transferred into a 1 cm cuvette and mixed with 500 µl reagent 1 (4 ml phenolate [2.35 g phenol, 10 g tri-sodium citrate and 1 g sodium hydroxide dissolved in 80 ml water] mixed freshly with 1 ml sodium nitroprusside solution 1 mg/ml). Then 100 µl reagent 2 (a freshly prepared aqueous solution of sodium dichloroisocyanurate 4 mg/ml) was added and the reactions kept in the dark for 2 h prior measurement of the absorbance at 655 nm. Standards containing 0-8 mg/l ammonia (in form of ammonium sulfate) were used for establishing the calibration curve.

LITERATURE CITED

Kempf, M., Beuerle, T., Bühringer, M., Denner, M., Trost, D., von der Ohe, K, et al. (2008). Pyrrolizidine alkaloids in honey: risk analysis by gas chromatography-mass spectrometry. *Mol. Nutr. Food Res.* 52, 1193-1200. Doi: 10.1002/mnfr.200800051
